# The correlation between bone mineral density measured at the forearm and at the lumbar spine or femoral neck: a systematic review and meta-analysis

**DOI:** 10.1186/s12891-025-08376-7

**Published:** 2025-02-14

**Authors:** Mario Virgilio Papa, Chiara Ceolin, Cristina Simonato, Giulia Termini, Federica Vilona, Alessandro Ruggiero, Anna Bertocco, Chiara Curreri, Rocco Talomo, Alessandra Coin, Giuseppe Sergi, Marina De Rui

**Affiliations:** 1 Geriatrics Division, Department of Medicine (DIMED), University of Padua, via Giustiniani 2, Padova, 35128 Italy; 2 Department of Neurobiology, Care Sciences and Society, Aging Research Center, Karolinska Institutet and Stockholm University, Stockholm, Sweden; 3 Research Unit of Anesthesia and Intensive Care, Department of Medicine, Campus Bio-Medico University, Rome, Italy

**Keywords:** Densitometry, Forearm, Bone mineral density, Osteoporosis, T-score, Older adults

## Abstract

**Background:**

Current guidelines for osteoporosis diagnosis do not recommend forearm dual-energy X-ray absorptiometry (DXA) as a standard tool, except in specific cases. This systematic review and meta-analysis investigates the potential correlation between forearm BMD and BMD at the lumbar and/or hip sites.

**Methods:**

The protocol was registered in PROSPERO (CRD42024568756), and the study adhered to the PRISMA guidelines. Major databases were systematically searched from their inception until August 2024 to identify studies evaluating the ability of forearm DXA scans to detect osteoporosis, particularly in comparison to central sites like the femoral neck and lumbar spine. A meta-analysis was conducted on studies that reported correlation coefficients between these measurements. Quality assessment was conducted independently by 3 reviewers following Quality Assessment of Diagnostic Accuracy Studies (QUADAS-2) criteria. Additionally, a narrative synthesis of the main findings across different patient groups was performed.

**Results:**

Thirteen studies were included. Published between 1992 and 2023, these studies involved 5941 participants. Forearm scans exhibited strong correlations with lumbar and femoral sites (pooled effect size 0.603, 95%CI 0.579–0.627 and 0.641, 95%IC 0.600–0.680, respectively) demonstrating good predictive value for central osteoporosis. Despite some result variations, forearm DXA scanning emerged as a valid method, especially when lumbar and femoral measures are challenging.

**Conclusions:**

A DXA scan of the distal forearm proves to be a valuable supplementary tool for identifying osteoporotic conditions. This could be particularly relevant in older patients, where conducting lumbar or hip scans is often challenging or not feasible.

**Supplementary Information:**

The online version contains supplementary material available at 10.1186/s12891-025-08376-7.

## Introduction

Osteoporosis is the most common metabolic bone disease in older adults and its prevalence is destined to increase along with aging societies [1]. It is defined by altered bone quantity, i.e. a decrease in bone mineral density (BMD) and quality [1]. Fractures are the most concerning complication of osteoporosis, damaging especially hip, spine, forearm and shoulder sites [1]. Fractures are associated with poor outcomes in older people: hip fractures significantly reduce functional performance, especially in the first six months after hospitalization, with important consequences not only for physical autonomy but also for survival, with mortality rates of 14 to 36% within 1 year of injury [2]. For these reasons, early detection of osteoporosis is essential to prevent bone fractures and to initiate preventive strategies, which include osteoporosis therapies and appropriate nutritional supplementation and muscular training for falls prevention [2, 3]. According to the most recent guidelines, dual-energy X-ray absorptiometry (DXA) is the gold standard for diagnosing osteoporosis [4]. DXA scans of lumbar spine and femur (total and neck) sites are performed to estimate density by measuring areal BMD, returning scores that are a number of standard deviations above or below the mean normal value of bone mineral density for young adults (T-score) [5]. The International Osteoporosis Foundation (IOF) defines osteoporosis as a lumbar or femur T-score ≤–2.5 [5].

Although not widely used, forearm BMD measurements have been validated for osteoporosis diagnosis and follow-up [6]. The guidelines for detecting osteoporosis recommend forearm BMD measurement where hip and/or spine analyses cannot be carried out for any reason, in cases of hyperparathyroidism, and where the patient is very obese (too heavy for the DXA table) [6]. Some authors suggest using forearm BMD measurement as a pre-screening tool for the general population [7], although it is not unanimously agreed that forearm DXA is a complete surrogate for central BMD assessment, which means that estimates of fracture risk using this peripheral segment may not always be reliable [8].

Consequently, the current systematic review and meta-analysis aims to provide a comprehensive understanding of the ability of forearm DXA scans to detect osteoporosis, regardless of the patients’ age.

## Methods

### Systematic review tool

This review adheres to PRISMA (http://www.prisma-statement.org/) and Meta-Analysis of Observational Studies in Epidemiology guidelines [9] and the protocol was registered on Prospero, CRD42024568756.

### Search strategy and selection criteria

The Embase Ovid, Scopus, PubMed, Cochrane Library, and Web of Science databases were searched for the terms “Forearm”, “Dual Energy X Ray Absorptiometry”, and “Osteoporosis”, from any date to August 2024. Only papers and reviews in English were selected. The articles of interest were studies of individuals with osteoporosis of any population, age or gender. References cited in the selected papers were inspected to identify any other potential articles. The abstract titles and full-texts were screened independently by three authors (C.S., G.T., F.V.). Any disagreements were solved by consulting the senior authors (M.V.P., C.C., M.D.R.).

### Inclusion and exclusion criteria

Inclusion criteria were: (1) diagnosis of osteoporosis; (2) comparison of forearm DXA scanning with lumbar and/or femur scanning. Exclusion criteria were: (1) case reports, abstracts, letters, and editorials; (2) studies not written in English; (3) animal model studies.

### Risk of bias assessment

Quality assessment was conducted independently by 3 reviewers (A.B., C.C., A.R.) following Quality Assessment of Diagnostic Accuracy Studies (QUADAS-2) criteria, as suggested by the Cochrane guidelines for diagnostic test accuracy reviews [10, 11]. The QUADAS-2 tool consists of 4 key domains that concern patient selection, index tests, reference standards, and patient flow. Each domain is assessed in terms of the risk of bias (low, high, or unclear), while the first 3 domains are also assessed in terms of applicability (low, high, or unclear).

### Data extraction

Data were extracted by four authors (C.S., G.T., F.V., M.V.P.). For each included study, the following information was recorded: (1) study design; (2) sample size, including number of female patients; (3) median/mean age of participants; (4) BMD of lumbar, femoral neck, and total hip sites; (5) T-scores of lumbar, femoral neck, and total hip sites; (6) Pearson’s correlations between forearm and lumbar/femur sites; (7) relevant findings.

### Data analysis

A meta-analysis was performed for data that were sufficiently homogenous in terms of statistical and methodological characteristics. The meta-analysis was performed using two different software: StatsDirect statistical software (http://www.statsdirect.com. England: StatsDirect Ltd 2024) and Comprehensive Meta-Analysis Version 4, focusing on the correlation coefficients (R) reported in the studies between forearm DXA scans and other sites. To calculate the effect size, these correlation coefficients were transformed into Z values using Fisher’s transformation. Statistical significance was defined as a p-value < 0.05. Heterogeneity among studies was assessed using the Q test, I², tau², and tau. I² values were used to classify heterogeneity as follows: <25% indicating low, 25–50% indicating moderate, and > 50% indicating high heterogeneity [12]. A fixed-effect model was applied when heterogeneity was low, while a random-effects model was used when heterogeneity was moderate or high. In the supplementary file, we reported the entire meta-analysis process and the funnel plot with the publication bias.

## Results

**Fig. 1:**
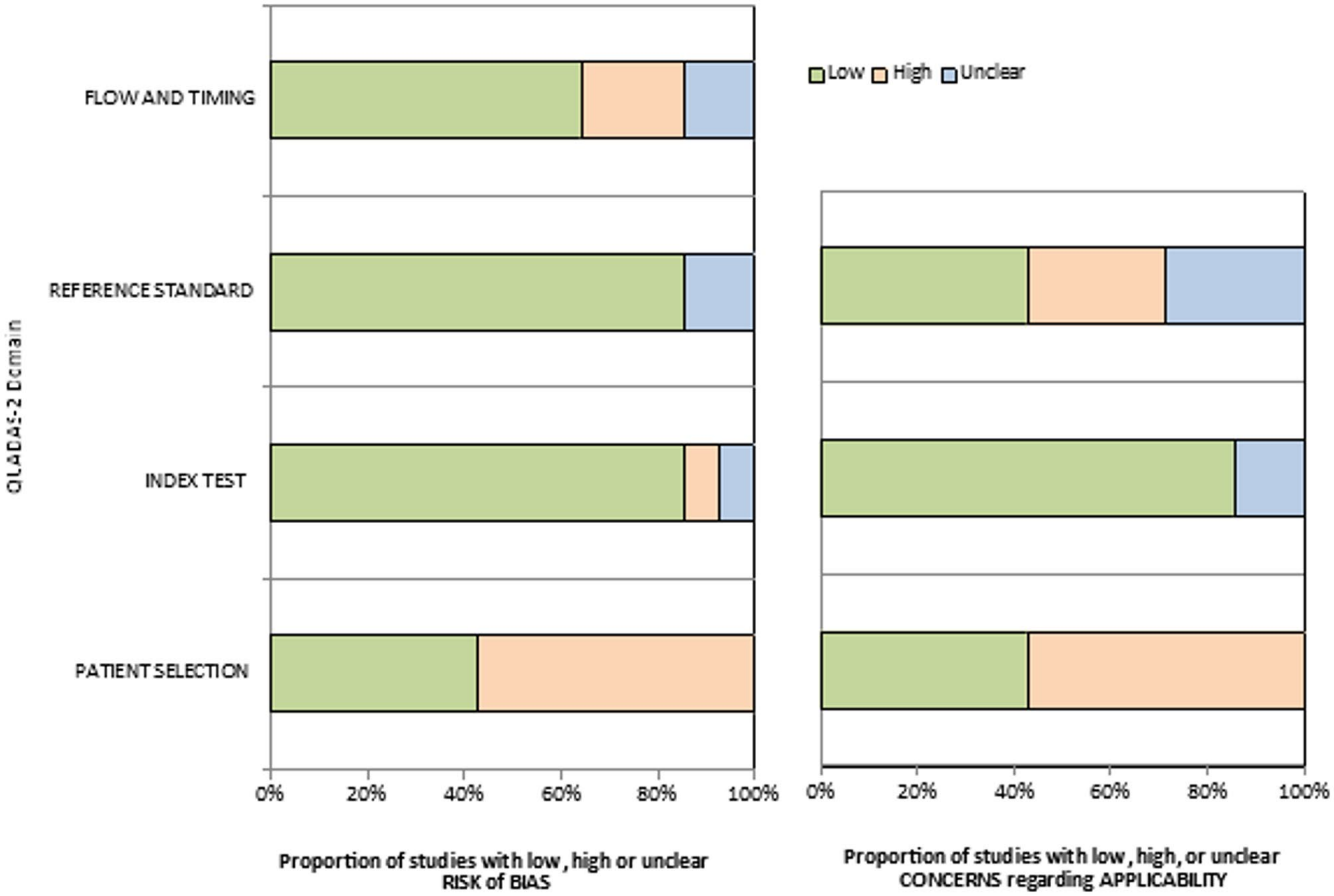
Applicability and risk of bias of the selected studies. *Notes*: The term “index test” refers to the forearm BMD measurement and describes how this test is conducted and interpreted. “Flow and timing” refers to the time interval between the index test (forearm BMD measurement) and the reference standard (spine and femur BMD measurements). Specifically, “flow” describes the order in which the BMD measurements are taken (forearm first, followed by spine and femur), while “timing” refers to the time interval between the index test (forearm BMD) and the reference tests (spine and femur BMD), when applicable

A total of 3365 studies were identified from the database searches, of which 2497 duplicates were excluded. After reviewing the titles and abstracts, 841 studies were discarded because they did not conform to the inclusion criteria; of the remaining 27 a further 11 were discarded (inappropriate populations, missing data, etc.) leaving 16 papers, the full manuscripts of which were assessed for eligibility. A further 3 papers were discarded as ineligible, leaving 13 studies for systematic review (please see Supplementary Fig. 1). The quality assessments of these 13 studies are reported in detail in Table 1. Five studies complied with all the QUADAS-2 items related to the risk of bias [13–17]; 3 studies had a low risk of bias in 3 of the 4 domains [18–20], and 2 studies had a low risk of bias in 2 of the 4 domains [21, 22]. No studies were considered to have a high risk of bias in 3 or 4 domains (Fig. 1).

**Table 1:** Quality Assessment of Diagnostic Accuracy studies (QUADAS-2)

Study	Risk of bias				Applicability concerns		
	Patient selection	Index test	Reference standard	Flow and timing	Patient selection	Index test	Reference standard
Walker 2019	☺	☺	☺	☺	☺	☺	?
Bouxsein 1999	☺	?	☺	☺	☺	?	?
Jones 1998	☹	☺	☺	☺	☹	☺	☺
Wood 2012	☹	☺	?	☹	☹	☺	?
Gautam 2022	☺	☺	☺	☺	☺	☺	☺
Mulder 2000	☹	☺	☺	?	☹	☺	☹
Sang Beom Ma 2023	☺	☺	☺	☺	☺	☺	☺
Pouillès 2001	☺	☺	☺	☺	☺	☺	☺
Picard 2004	☹	☺	?	?	☹	☺	?
Melton 2005	☹	☺	☺	☹	☹	☺	☹
Uz Zaman 2013	☺	☺	☺	☺	☺	☺	☺
Rosenthall 2002	☹	☺	☺	☺	☹	☺	☺
Ryan 1992	☹	☺	☺	☹	☹	☺	☹

☺Low Risk ☹High Risk? Unclear Risk

### Study characteristics

Of the 13 papers selected, 11 were observational studies [13–24], and 1 were randomized trials [25]. All the studies dealt with osteoporosis and its diagnosis by DXA scan, and met the aforementioned inclusion criteria. A total of 5941 participants were involved in the included studies, which were carried out in Canada [22, 23], the USA [13, 18, 20, 21, 25], India [14], South Korea [17], France [15], Pakistan [16] and the UK [19, 24], and were published between 1992 and 2023. In all the articles BMD (g/cm^2^) was measured by DXA scanning.

Table 2 lists all the selected studies and the main results regarding any positive or negative associations between forearm and lumbar/femur scans.

**Table 2:** Selected studies investigating the associations between forearm DXA and lumbar and femoral sites

Author/year/ reference	Study design	Country	Sample size (*N* females)	Mean/ median age	BMD g/cm^2^ L	BMD g/cm^2^ FN	BMD g/cm^2^ TH	BMD g/cm^2^ F	T-score L	T-score FN	T-score TH	T-score F	*r* F-L	*r* F-FN	*r* F-TH	Findings
Sang Beom Ma 2023 [17]	Retrospective	South Korea	456 (456)	60.6	0.96	0.75	0.75	0.5	-1.46	-1.62	-1.63	-1.89	-	0.6	0.61	A distal forearm DEXA scan performed in addition to a central DEXA may be an effective tool for detecting the osteoporotic conditions of the distal radius, which is associated with an increased risk of osteoporotic Distal Radius Fractures
Walker, 2020 [12]	Cross-Sectional	USA	721 (68.4% females) celiac patients	43.6 (16.2)	-	-	-	-	-0.9	-0.9	-0.6	-0.7	-	-	-	Osteoporosis screening of patients with celiac disease should include measurement of the distal radius in addition to the hip and lumbar spine.
Gautam, 2022 [13]	Cross-Sectional	India	352 post-menopausal women	60.7 (6.8)	0.802	0.614	-	0.549	-2.2	-2.1		-2.3	0.62	0.65	-	Forearm BMD may be predictive of trabecular microarchitecture and central site osteoporosis at the femoral neck and lumbar spine in postmenopausal women.
Pouillès, 2001 [14]	Prospective	France	234 healthy women	45–60	-	-	-	-	-	-	-	-	0.56	-	0.43	Forearm DXA measurement as a prescreening tool in early postmenopausal women should directly identify about 50% of women without axial osteoporosis
Zaman, 2013 [15]	Prospective	Pakistan	279 (F/M: 256/23)	63.25 (10.62)	0.834	0.632	0.767	0.456	-1.257	-1.564	-1.909	-2.004	-	-	-	Combining distal forearm with spine and hip BMD can identify more patients with low bone mass or osteoporosis.
Bouxsein, 1999 [16]	Randomized Trial	USA	120 post-menopausal women	70.2 (4.8)	0.886	0.647	0.774	0.489	-	-	-	-	0.56	0.57	0.58	Early changes in forearm BMD in elderly women on alendronate therapy do not predict longer term changes in BMD at hip and spine.
Rosenthall, 2002 [17]	Prospective	Canada	1300 women	58.3 (13.9)	-	-	-	0.279	-	-	-	-	0.6	0.65	0.67	The use of peripheral DXA substantially reduces the number of false negatives, compared to standard projections.
Jones, 1998 [18]	Cross-Sectional	UK	422 women	60.9 (12.3)	0.311	0.221	-	-	-	-	-	-	0.64	0.7	-	Positive correlations between distal forearm BMD and lumbar spine and femoral neck BMD.
Melton III, 2005 [19]	Epidemiological Study	USA	699 (F/M = 351/348)	22–90	F 0.93/ M 0.91	F 0.70/ M 0.60	F 0.74/ M 0.66	F 0.71/ M 0.59	-	-	-	-	-	-	-	Strong correlations between forearm BMD and corresponding regions from whole body scans, but different estimates of osteoporosis prevalence and fracture risk.
Mulder, 2000 [20]	Observational	USA	123 post-menopausal women	64.6 (42–82)	-	-	-	-	-	-	-	-	0.536	0.547	0.583	Inconsistencies in the diagnosis of osteoporosis when a single site is measured. Measuring multiple sites improves diagnosis of low BMD.
Ryan, 1992 [22]	Retrospective	UK	100	29–69	0.962	0.763	-	0.527	-	-	-	-	0.561	0.554	-	Significant correlations between axial BMD and all forearm sites. Forearm BMD can be used to diagnose osteoporosis and may also have a wider role in predicting fractures.
Picard, 2004 [23]	Prospective	Canada	835 women	20–85	-	-	-	0.27	-	-	-	-1.59	0.683	0.712	-	Forearm DXA can be a useful tool for diagnosing osteoporosis in areas where central DXA is problematic.
Wood, 2012 [24]	Retrospective	USA	300 (F/M = 257/43)	64 (12)	-	-	-	-	-0.9	-	-	-1.3	-	-	-	Distal forearm BMD should be assessed in all patients with a diagnosis of PHPT in addition to standard DXA projections.

*Abbreviations*: L = lumbar; FN = Femoral Neck; TH = Total Hip; F = Forearm; r = Pearson’s correlation

### Description of the included studies

In 721 patients with celiac disease (mean age 43.6 years), the prevalence of osteoporosis at the forearm site was 11.5% (compared to 12% at the lumbar spine and 5.3% at the total hip). A greater degree of villous atrophy at diagnosis was associated with male sex and lower T- and Z-scores, but only at the radius [13]. Furthermore, in a separate cohort of 300 patients (257 females) with a mean age of 64 years undergoing parathyroidectomy, the lowest T-scores were observed at the peripheral site [21], suggesting that distal radius DXA scans could be valuable for osteoporosis screening in patients with both celiac disease and hyperparathyroidism [13, 21].

Gautam et al.‘s cross-sectional study of 352 postmenopausal women (mean age 60.7 years) found that those with forearm osteoporosis were more likely to have central osteoporosis. This suggests that forearm scans could be useful for predicting osteoporosis at central sites, such as the femur and lumbar spine, and for assessing trabecular microarchitecture in postmenopausal women [14]. The authors reported a significant positive correlation between all segments of the distal forearm and Trabecular Bone Score (TBS) (*r* = 0.4, *p* < 0.001). Forearm BMD (specifically at the distal third, mid-distal radius, and ultra-distal radius) showed strong correlations with BMD at the lumbar spine and femoral neck. Notably, patients with osteoporosis at the mid-distal radius were more likely to have osteoporosis at the femoral neck and lumbar spine compared to those with osteoporosis at the other forearm sites [14]. Analyses of the receiver operating characteristic (ROC) curves showed that T-scores at all segments of the forearm were good predictors of osteoporosis at the central sites, whether combined or at the femoral neck and lumbar spine individually (Area Under Curve-AUC > 0.800, *p* < 0.001 for all) [14]. Pouillès et al. measured BMD at the proximal and distal radius (by peripheral DXA-pDXA) and at the lumbar and femur sites (by DXA) in 234 healthy women aged 45–60 years. The correlations between the axial and peripheral sites were moderate (r values ranging from 0.4 to 0.6) [15], while the correlation between the lumbar spine and forearm BMD (*r* = 0.53–0.60) was better than between the hip and forearm BMD (*r* = 0.39–0.47). All the women were identified as osteoporotic at the forearm scan, 39% also had lumbar/hip osteoporosis (T-score ≤-2.5), and 58–62% had T-scores between − 1 and − 2.5. Only one person with peripheral osteoporosis was classified as normal in the axial scans [15]. At the ROC analyses, the proximal and distal radius measurements had comparable ability to identify women with axial osteoporosis (AUC = 0.75 and AUC = 0.80, respectively, *p* < 0.001). Similarly, Rosenthall’s Canadian study of 1,300 women (mean age 58.3 years) found moderate correlations between the peripheral and axial sites (r values ranging from 0.51 to 0.67) [23]. The results of the ROC analyses of lumbar spine and femoral neck osteoporosis showed an AUC of 0.854 for the distal forearm, and 0.850 for the proximal forearm [23]. Finally, Jones et al. reported good Pearson’s correlations between distal forearm BMD and lumbar spine (*r* = 0.64) and femoral neck (*r* = 0.70) BMD in 422 women, mean age 60.9 years [24]. The ROC curves describing the effectiveness of distal forearm BMD in identifying central osteoporosis showed an AUC of 0.82 [24]. All four studies proposed using forearm T-scores as a “pre-screening” tool to refer perimenopausal women to a more comprehensive axial evaluation, and agreed that peripheral scans could be helpful in reducing the number of false negatives, i.e. patients who would otherwise be candidates for treatment [14, 15, 23, 24]. Confirming these results, Picard and colleagues, as well as Ryan and colleagues, considered peripheral BMD measurement together with good clinical evaluation of a patient’s risk of osteoporosis to be a valid tool for diagnosing this osteo-metabolic disease when axial scans are not available [22]. Their conclusions were based on assessment of, respectively, 835 and 100 women (aged 20–85 years) by central (lumbar and femur) and peripheral (forearm) DXA [19, 22]. The strongest correlation coefficients between the peripheral and axial sites were for the ultra-distal radius [19] and the distal radius [22]. Ryan et al. reported moderate correlations between forearm and, especially, lumbar spine values (*r* = 0.67), and between forearm and femoral neck values (*r* = 0.57) (both *p* < 0.001) [19], while Picard et al. found a higher correlation between forearm and femoral neck (*r* = 0.71; between forearm and lumbar spine: *r* = 0.68; *p* < 0.001) [22]. In the USA, Mulder and colleagues performed DXA scans of lumbar spine, total hip, femur neck and forearm in 123 postmenopausal women (aged 42–82 years), and obtained correlations between the one-third radius and the lumbar (*r* = 0.54) and total hip sites (*r* = 0.58) [18].

According to Zaman and colleagues, performing DXA scans of the distal forearm in addition to the hip and lumbar sites improves the diagnosis of osteoporosis [16]. In their study, carried out in Pakistan, they assessed 279 consecutive patients (256 females), mean age 63 years, and found that combining distal forearm BMD with T-scores increased the diagnosis of osteoporosis from 26 to 35% [16].

Finally, in a study of 456 individuals who underwent DEXA scans at three sites (lumbar spine, proximal femur, and distal forearm), the correlation coefficient between BMD measured at the forearm and at the total hip was *r* = 0.61, and between BMD at the forearm and the femoral neck was *r* = 0.6 (*p* < 0.001 for both) [17].

#### Meta-analyses results: forearm scan vs. lumbar spine

**Fig. 2:**
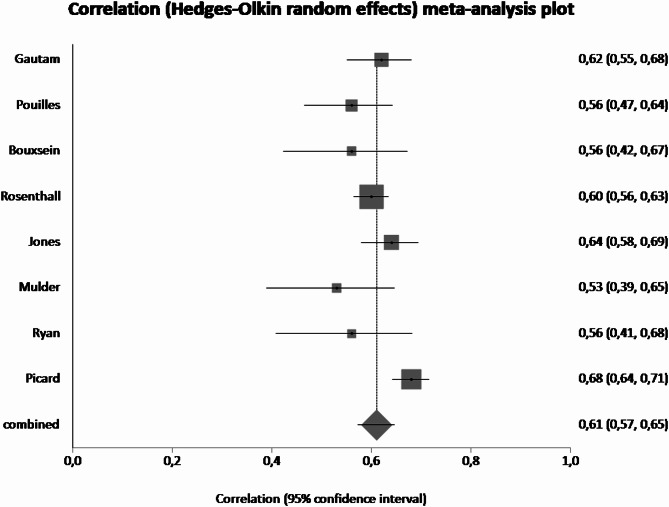
Forest plot comparing BMD measures at the forearm scan and the lumbar site

We assessed eight studies [14, 15, 18, 19, 22–25] comparing the effectiveness of forearm scan to lumbar spine scans for diagnosing osteoporosis. The pooled effect size was *r* = 0.603 with a 95% confidence interval (CI: 0.579 to 0.627). The I² statistic indicated 0% heterogeneity, reflecting a high level of consistency across the studies. In other words, we found a strong correlation between forearm scan and lumbar spine. In Fig. 2 is reported the correlation between Forearm scan and Lumbar spine.

#### Meta-analyses results: forearm scan vs. femoral neck

**Fig. 3:**
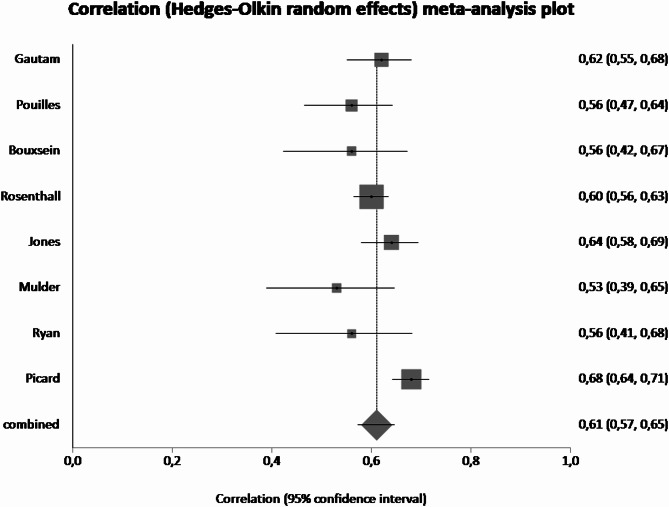
Forest plot comparing BMD measures at the Forearm scan and at the Femoral Neck

In a second analysis involving eight studies [14, 17–19, 22–25], a random-effects model was used. The pooled effect size was *r* = 0.641 (95% CI: 0.600 to 0.680). The I² statistic was 71%, indicating that 71% of the observed variance was due to true effect differences rather than sampling error. We found a strong correlation between forearm scan and femoral neck. In Fig. 3 is reported the correlation between Forearm scan and Femoral Neck.

## Discussion

This systematic review and meta-analysis aimed to assess the correlation between forearm BMD and BMD at the lumbar and/or hip sites. Considering the existing heterogeneity in the available evidence, our study aimed to address the knowledge gap on this topic through a systematic analysis. Despite the variability among studies, our results reveal a strong correlation between BMD measured at the forearm and at other key sites of densitometric analysis in adults.

Given the increasing aging of the population and consequently the overall progressive increase of osteoporosis prevalence, in recent decades, there has been a proliferation of technology for measuring axial and peripheral BMD [26]. In fact, distal radius fractures are a significant concern, accounting for 37% of all osteoporotic fractures and becoming one of the most considerable expenses that healthcare systems will face [17]. Although less dangerous in terms of outcomes compared to hip fractures, distal radius fractures are considered major osteoporotic fractures, as well as vertebral and hip fractures. They occur approximately 15 years before hip fractures and are therefore regarded as predictors of subsequent osteoporotic fractures [27, 28]. A history of forearm fractures in women increases the risk of hip fractures by 1.4 times and the risk of vertebral fractures by 5.2 times compared to those without peripheral fractures. Consequently, early detection of radius fracture risk may help identify patients at risk of vertebral or hip fractures. Peripheral DXA scanning for distal radius BMD appears to be more effective for this purpose than central scans, particularly in terms of convenience and feasibility. However, distal forearm BMD screening by DXA is not currently prioritized [17].

In the following sections, we will analyze the potential clinical implications of our study results, with a particular focus on older adults, who represent the population most frequently affected by osteoporosis. This group could benefit from a quicker and more accessible assessment of fracture risk.

### The usefulness of peripheral scans: possible clinical implications

The measure of BMD at central scans, i.e. hip and spine, is considered the gold standard for diagnosing osteoporosis and assessing fracture risk, and for monitoring the treatment effect. However, the results of this meta-analysis suggest a strong correlation between central and peripheral BMD measurements, supporting and reinforcing the idea of using BMD not only as a screening tool but also as a potential diagnostic tool, especially in certain patient groups. Peripheral methods offer several advantages over axial analysis techniques. Their portability and low cost allow for large-scale measurements, though they are limited to assessing only one or, at most, two skeletal sites [26]. Forearm scans, in particular, are highly versatile, especially in cases where central scans, such as those of the lumbar spine and femur, are challenging to perform. This is especially relevant for individuals with certain medical conditions, such as severe arthritis, obesity, or previous spine surgery, where traditional central scans may show artifact-induced increases in BMD [15]. Moreover, the progressive increase in fat mass associated with aging can lead to larger measurement errors and reduced reproducibility [29–31]. Spine and hip BMD measurements are also affected by degenerative changes, which are common in older adults. These factors, combined with the critical need for proper positioning and image analysis in older patients, can result in falsely elevated BMD values and underestimate fracture risk [32]. Additionally, reduced mobility, including impaired walking, gait speed, and balance due to muscle loss, shifts in center of gravity, neurodegenerative conditions, and overall aging, often complicates correct positioning on the examination table. In this context, forearm scans may offer a more comfortable and accessible alternative [18, 33]. The distal third of the radius is currently regarded as the most reliable site for predicting osteoporosis [28, 34, 35]. This region’s predictive accuracy is linked to the physiological mechanism of forearm fractures: the metaphysis of the distal radius, which includes both cortical and trabecular bone, is more susceptible to age-related changes, leading to a greater loss of spongy bone [17]. This results in earlier loss of BMD in the ultradistal radius compared to other sites. Nevertheless, the authors conclude that both distal radius and femur scans provide the best predictions for forearm fracture risk [17].

Finally, another notable advantage is the minimal radiation exposure, which is of particular concern for patients and healthcare professionals aiming to minimize radiation risks. The radiation dose from including the distal forearm in a standard DXA examination is 0.1 microsieverts (µSv), which is negligible compared to the average annual effective dose of natural background radiation, approximately 2–4 mSv/year [36].

### Limitations

Despite the benefits, several limitations must be addressed. A major limitation is that only English-language publications were included, and gray literature was not considered, potentially excluding relevant studies. Additionally, most research focused on higher-income populations, which may not be representative of the global population, with few studies conducted in low- and middle-income countries (LMICs). This may limit the generalizability of the findings. One important limitation of our study is that we were unable to assess the predictive ability of forearm BMD for fracture risk. Given that the primary aim of treatment is to reduce fracture risk rather than simply addressing DXA scan results, this represents a significant gap in our analysis. Future research should aim to evaluate the role of forearm BMD in predicting fractures, which would help to better understand its clinical utility in fracture risk assessment.

Several open questions remain. For instance, could a standardized method be crucial for maintaining the quality and reliability of results? Is there a need for standardized protocols to ensure that forearm scans produce consistent results across various healthcare settings? The lack of robust evidence highlights the necessity for further research, particularly randomized trials and longitudinal studies, to assess whether forearm projections could effectively replace standard projections.

## Conclusion

We demonstrated a strong correlation between BMD measurements in standard projections and those in the forearm. Performing a DXA scan of the distal forearm, in addition to the standard DXA views of the lumbar spine and hip, could serve as an additional tool for identifying osteoporotic conditions at the distal radius, a site associated with a higher risk of osteoporotic fractures. Geriatricians and other specialists evaluating older adults should actively consider utilizing forearm DXA scans, particularly in instances where traditional sites like the lumbar spine and femur are compromised or regarded as unreliable. T-scores of the ultradistal radius may be used to complement lumbar and hip scans in cases of uncertainty, but further studies are needed across different subgroups with various secondary osteoporosis conditions to validate the findings discussed here.

## Electronic supplementary material

Below is the link to the electronic supplementary material.

Supplementary Material 1

Supplementary Material 2

Supplementary Material 3

Supplementary Material 4

## Data Availability

Data is provided within the manuscript or supplementary information files.
