# Supplementary material for: The correlation between bone mineral density measured at the forearm and at the lumbar spine or femoral neck: a systematic review and meta-analysis
Source: BMC Musculoskelet Disord. 2025 Feb 14;26:151. doi: 10.1186/s12891-025-08376-7 (PMC11827141; doi:10.1186/s12891-025-08376-7)
Supplement: Supplementary file 2 — Supplementary Material 2 [file 12891_2025_8376_MOESM2_ESM.rtf]

!!help!-> 1159 <-!help!! 
Correlation meta-analysis

Study	Size	Correlation	Approximate 95% CI		
1	352	0,62	0,551191	0,680433	Gautam	
2	234	0,56	0,465161	0,64213	Pouilles	
3	120	0,56	0,423242	0,671808	Bouxsein	
4	1.300	0,6	0,564031	0,633697	Rosenthall	
5	422	0,64	0,579974	0,693114	Jones	
6	123	0,53	0,389513	0,646385	Mulder	
7	100	0,56	0,408516	0,681461	Ryan	
8	835	0,68	0,641762	0,714865	Picard	

Stratum	Standardized effect	Variance	% Weights (fixed, random, size)		
1	0,725005	0,002865	10,080878	13,781017	10,097533	Gautam	
2	0,632833	0,004329	6,672444	11,256978	6,712565	Pouilles	
3	0,632833	0,008547	3,379549	7,368051	3,442341	Bouxsein	
4	0,693147	0,000771	37,463894	20,290917	37,292025	Rosenthall	
5	0,758174	0,002387	12,102831	14,871566	12,105565	Jones	
6	0,590145	0,008333	3,466205	7,499293	3,528399	Mulder	
7	0,632833	0,010309	2,801849	6,438715	2,868617	Ryan	
8	0,829114	0,001202	24,032351	18,493463	23,952955	Picard	

Hedges-Olkin fixed effects
Pooled correlation = 0,620355 (95% CI = 0,599439 to 0,640424)
Z (test correlation differs from 0) = 42,692341  P < 0,0001
Non-combinability of studies
Cochran Q = 16,756983  (df = 7)  P = 0,019
Moment-based estimate of between studies variance = 0,003663
I² (inconsistency) = 58,2% (95% CI = 0% to 79,1%) 
Hedges-Olkin random effects
Pooled correlation = 0,61037 (95% CI = 0,572158 to 0,645938)
Z (test correlation differs from 0) = 23,655586  P < 0,0001
Bias indicators
Begg-Mazumdar: Kendall's -0,214286  P = 0,3988
Egger: bias = -1,634385894033155 (95% CI = -4,640505679964632 to 1,3717338918983222)  P = 0,2317
Schmidt-Hunter
Weighted mean correlation (95% CI): 0,618345 (0,588849 to 0,647841)
Z (test correlation differs from 0) = 41,08848 P < 0,0001
Observed variance across studies: 0,001812
Variance due to sampling error: 0,000877
Variance in the population correlations: 0,000934
95% Credibility interval for weighted mean correlation: 0,558436 to 0,678254
Indicators of homogeneity/heterogeneity:
1.	Residual standard deviation (should be smaller than 1/4 WMC: 0,154586): 0,030566	
2.	Percent of observed variance accounted for by sampling error (should be at least 75%): 48,432134	
3.	Chi-square test of heterogeneity: 16,517959 P = 0,0208	


!!help!-> 1159 <-!help!! 


!!help!-> 1159 <-!help!! 
Correlation meta-analysis

Study	Size	Correlation	Approximate 95% CI		
1	456	0,6	0,537803	0,655701	Sang Beom Ma	
2	352	0,65	0,585233	0,706526	Gautam	
3	120	0,57	0,435224	0,679789	Bouxsein	
4	1.300	0,65	0,617452	0,680326	Rosenthall	
5	422	0,7	0,64783	0,745635	Jones	
6	123	0,547	0,409634	0,660111	Mulder	
7	100	0,55	0,396406	0,673644	Ryan	
8	835	0,71	0,674653	0,742098	Picard	

Stratum	Standardized effect	Variance	% Weights (fixed, random, size)		
1	0,693147	0,002208	12,296417	14,517745	12,297735	Sang Beom Ma	
2	0,775299	0,002865	9,473398	13,42046	9,492988	Gautam	
3	0,647523	0,008547	3,175896	8,119781	3,236246	Bouxsein	
4	0,775299	0,000771	35,206298	17,673197	35,059331	Rosenthall	
5	0,867301	0,002387	11,373507	14,201557	11,380798	Jones	
6	0,61409	0,008333	3,257329	8,24221	3,317152	Mulder	
7	0,618381	0,010309	2,633008	7,233615	2,696872	Ryan	
8	0,887184	0,001202	22,584148	16,591435	22,518878	Picard	

Hedges-Olkin fixed effects
Pooled correlation = 0,656984 (95% CI = 0,638239 to 0,67495)
Z (test correlation differs from 0) = 47,797367  P < 0,0001
Non-combinability of studies
Cochran Q = 23,888846  (df = 7)  P = 0,0012
Moment-based estimate of between studies variance = 0,005838
I² (inconsistency) = 70,7% (95% CI = 22,8% to 84,1%) 
Hedges-Olkin random effects
Pooled correlation = 0,64107 (95% CI = 0,599908 to 0,678847)
Z (test correlation differs from 0) = 22,237111  P < 0,0001
Bias indicators
Begg-Mazumdar: Kendall's -0,285714  P = 0,2751
Egger: bias = -2,464140304142729 (95% CI = -5,9350519144431555 to 1,0067713061576975)  P = 0,133
Schmidt-Hunter
Weighted mean correlation (95% CI): 0,65435 (0,621756 to 0,686944)
Z (test correlation differs from 0) = 39,347709 P < 0,0001
Observed variance across studies: 0,002212
Variance due to sampling error: 0,000707
Variance in the population correlations: 0,001505
95% Credibility interval for weighted mean correlation: 0,578304 to 0,730397
Indicators of homogeneity/heterogeneity:
1.	Residual standard deviation (should be smaller than 1/4 WMC: 0,163588): 0,0388	
2.	Percent of observed variance accounted for by sampling error (should be at least 75%): 31,955366	
3.	Chi-square test of heterogeneity: 25,034919 P = 0,0007	


!!help!-> 1159 <-!help!! 
