# Supplementary material for: The correlation between bone mineral density measured at the forearm and at the lumbar spine or femoral neck: a systematic review and meta-analysis
Source: BMC Musculoskelet Disord. 2025 Feb 14;26:151. doi: 10.1186/s12891-025-08376-7 (PMC11827141; doi:10.1186/s12891-025-08376-7)
Supplement: Supplementary file 4 — Supplementary Material 4 [file 12891_2025_8376_MOESM4_ESM.docx]

**Supplementary Figure 1. PRISMA flow diagram of the process of (and reasons for) including and excluding studies.**

**Identification of studies via databases and registers**

Articles removed *before screening*:

Duplicate articles removed (n = 2497)

Articles marked as ineligible by automation tools (n = 0)

Articles removed for other reasons (n = 0)

Articles identified from:

Embase Ovid (n= 1028)

Scopus (n= 996)

PubMed (n= 868)

Cochrane Library (n=110)

Web Of Science (n=363)

**Identification**

Articles excluded (n = 841):

Not in English (n=5)

No outcome reported (n=713)

Unsuitable population (n=123)

Articles screened

(n = 868)

Articles sought for retrieval

(n = 27)

Full text not retrievable (n = 11)

**Screening**

Full-text articles excluded due to DXA not having been used for diagnosis (n = 3).

Articles assessed for eligibility

(n = 16)

Total number of Articles included in the systematic review

(n = 13)

**Included**
